# Anti-NASH Drug Development Hitches a Lift on PPAR Agonism

**DOI:** 10.3390/cells9010037

**Published:** 2019-12-21

**Authors:** Joost Boeckmans, Alessandra Natale, Matthias Rombaut, Karolien Buyl, Vera Rogiers, Joery De Kock, Tamara Vanhaecke, Robim M. Rodrigues

**Affiliations:** Department of In Vitro Toxicology & Dermato-Cosmetology (IVTD), Faculty of Medicine and Pharmacy, Vrije Universiteit Brussel (VUB); Laarbeeklaan 103, 1090 Brussels, Belgium; Joost.Boeckmans@vub.be (J.B.); Alessandra.Natale@vub.be (A.N.); Matthias.Rombaut@vub.be (M.R.); Karolien.Buyl@vub.be (K.B.); Vera.Rogiers@vub.be (V.R.); Joery.De.Kock@vub.be (J.D.K.); Tamara.Vanhaecke@vub.be (T.V.)

**Keywords:** non-alcoholic steatohepatitis (NASH), non-alcoholic fatty liver disease (NAFLD), peroxisome proliferator-activated receptor (PPAR), elafibranor, lanifibranor, saroglitazar, pioglitazone

## Abstract

Non-alcoholic fatty liver disease (NAFLD) affects one-third of the population worldwide, of which a substantial number of patients suffer from non-alcoholic steatohepatitis (NASH). NASH is a severe condition characterized by steatosis and concomitant liver inflammation and fibrosis, for which no drug is yet available. NAFLD is also generally conceived as the hepatic manifestation of the metabolic syndrome. Consequently, well-established drugs that are indicated for the treatment of type 2 diabetes and hyperlipidemia are thought to exert effects that alleviate the pathological features of NASH. One class of these drugs targets peroxisome proliferator-activated receptors (PPARs), which are nuclear receptors that play a regulatory role in lipid metabolism and inflammation. Therefore, PPARs are now also being investigated as potential anti-NASH druggable targets. In this paper, we review the mechanisms of action and physiological functions of PPARs and discuss the position of the different PPAR agonists in the therapeutic landscape of NASH. We particularly focus on the PPAR agonists currently under evaluation in clinical phase II and III trials. Preclinical strategies and how refinement and optimization may improve PPAR-targeted anti-NASH drug testing are also discussed. Finally, potential caveats related to PPAR agonism in anti-NASH therapy are stipulated.

## 1. Introduction

Non-alcoholic steatohepatitis (NASH) is an advanced form of non-alcoholic fatty liver disease (NAFLD) in which intrahepatic lipid accumulation in at least 5% of the hepatocytes is accompanied by hepatic inflammation and eventually fibrosis. NASH can further progress to life-threatening cirrhosis and hepatocellular carcinoma, and as such represents an emerging cause for liver transplantation. NAFLD is projected to affect 33.5% of the adult population by 2030, of which 27% will suffer from NASH. Currently, no effective therapies exist that can resolve NASH, yet considerable efforts have been made not only to understand the mechanisms by which this disease progresses but also to develop a suitable therapy [1,2,3].

The occurrence of NASH is strongly associated with the diagnosis of metabolic syndrome and obesity. As a consequence, introducing a balanced lifestyle seems to be a prime intervention when treating NASH. Weight loss of at least 10% of the body weight has been shown to solve NASH within one year [4]. In addition, bariatric surgery also proved to resolve NASH in 85% of obese patients who failed to lose weight through lifestyle modifications [5]. However, it was more efficient in patients with mild NASH than in those suffering from a more severe form of the disease. Intensive diets, lifestyle changes, or weight-loss surgery are often not attainable by the patients, and it has been shown that also a number of NASH patients is lean. These patients do not suffer from the metabolic syndrome, nor are they obese, but they do often carry specific genetic polymorphisms that have been associated with an increased propensity for developing NAFLD and NASH, such as PNPLA3 rs738409 [6]. Yet, the vast majority of NASH patients are obese and suffer from insulin resistance, type 2 diabetes, and hyperlipidemia. Therefore, drugs that target pathways disturbed in the metabolic syndrome are thought to hold anti-NASH properties as well. A major drug class administered to patients suffering from the metabolic syndrome targets peroxisome proliferator-activated receptors (PPARs). These multi-regulatory, ligand-activated, nuclear transcription factors regulate a multitude of processes that are mainly related to lipid metabolism, glucose homeostasis, and insulin signaling [7]. Therefore, PPARs are also attractive targets to tackle NAFLD. Distinct isotypes of PPARs can get activated by endogenous molecules (i.e., fatty acids and phospholipids) as well as synthetic ligands [8]. Synthetic PPAR-γ agonists, which are typically referred to as thiazolidinediones, sensitize the liver for insulin, and are indicated as a treatment for type 2 diabetes. Fibrates, which are agonists of PPAR-α, exert anti-hyperlipidemic effects. Fenofibrate was shown to additionally reduce the so-called ‘regulated on activation normal T-cell expressed and secreted’ (RANTES) serum levels in type 2 diabetes patients with hypertriglyceridemia [9], also indicating anti-inflammatory properties.

Nonetheless, up to now, none of the PPAR agonists on the market has demonstrated satisfactory efficacy in the resolution of NASH. Yet, PPAR agonists represent one of the most advanced classes of anti-NASH molecules currently in the pipeline of drug development [3,10,11].

This review discusses the application of PPAR agonists as a potential treatment of NASH. Based on the underlying molecular mechanisms of this therapy, possible caveats related to the administration of PPAR agonists are described. Furthermore, a novel strategy that potentially could improve PPAR-targeted preclinical anti-NASH drug testing is presented.

## 2. PPAR Tissue Distribution and Working Mechanism

The PPAR family consists of three isotypes, indicated by PPAR-α (*NR1C1*), -δ (syn. -β) (*NR1C2*), and -γ (*NR1C3*) [12,13]. PPAR-α was the first discovered isotype and was named after its ability to induce peroxisome proliferation in rodents [7]. All PPAR isotypes control lipid metabolism and are as such mainly expressed in tissues with high metabolic activity. Despite sharing high sequence homology and key functions, the PPAR isotypes are encoded by different genes, which are located on different chromosomes, and more or less specifically expressed in the body [14]. They regulate comparable as well as different processes and are activated by distinct synthetic ligands, resulting in the transcription of both overlapping and distinct downstream target genes [8,14]. PPAR-α and PPAR-δ are largely expressed in tissues with high mitochondrial and peroxisomal β-oxidative activity. PPAR-α is primarily expressed in liver, heart, kidneys, and brown adipose tissue, whereas PPAR-δ occurs ubiquitously. PPAR-γ is mainly expressed in white adipose tissue and is essential for adipocyte differentiation. It is also present in macrophages [8], including the liver-specific Kupffer cells.

**Figure 1:**
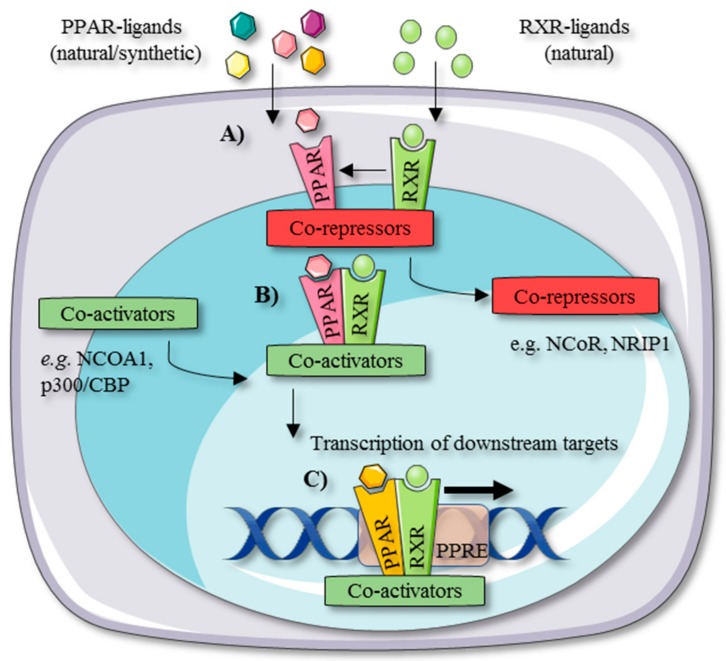
Mechanism of peroxisome proliferator-activated receptor (PPAR) activation and downstream gene transcription. (**A**) The PPAR receptor forms a heterodimer with a retinoid X receptor (RXR) upon the binding of an activating ligand. (**B**) PPAR-RXR heterodimerization causes the release of corepressors and recruitment of coactivators. (**C**) The PPAR-RXR heterodimer binds to PPAR-responsive elements on the DNA, resulting in the transcription of downstream target genes.

The PPAR isotypes share a similar working mechanism. Upon the binding of an activating ligand to a particular PPAR isotype, the PPAR concerned heterodimerizes with a retinoid X receptor (RXR) to bind specific peroxisome proliferator response elements (PPREs) on the DNA, resulting in the transcription of its downstream target genes (Figure 1) [8,15].

Coactivators (e.g., p300/CREB-binding protein (p300/CBP) and nuclear receptor coactivator 1 (NCOA1)) and corepressors (e.g., nuclear receptor co-repressor 1 (NCoR1) and nuclear receptor-interacting protein 1 (NRIP1)) tightly and selectively control these transcriptional inductions and repressions. This also lays at the structural basis of partial agonists that exhibit altered pharmacological properties [16,17,18,19]. Corepressors suppress the transcription of PPAR-related genes in the unliganded state. When a PPAR is activated by a specific ligand, coactivators translocate the corepressors, resulting in downstream transcription. Another mechanism by which PPARs modulate gene transcription is transrepression, which occurs independently from PPREs. Multiple molecular mechanisms have been described that regulate PPAR-mediated transrepression. In a ‘direct’ model of PPAR transrepression, PPARs bind to inflammatory transcription factors, such as nuclear factor-κB (NF-κB), activator protein-1 (AP-1), and signal transducer and activator of transcription (STAT), to prevent protein–protein interaction and therefore binding to response elements. Second, PPARs regulate the mitogen-activated protein kinase (MAPK) pathway. PPARs also interact with coactivators that regulate PPAR activation and repression. Yet, these coregulators are not specific to PPARs and therefore competition among coactivators can occur between PPARs and inflammatory transcription factors such as NF-κB and AP-1. Transrepression can occur as well through the action of corepressors on the promotor regions of inflammatory genes such as NF-κB, AP-1, and STAT 5–7. For example, PPAR-δ agonism in macrophages leads to the release of corepressors that will exert on their turn repressing activity on NF-κB, balancing the distribution of these cofactors. Furthermore, PPAR-α agonists induce the inhibitor of kappa B (IκB)α in hepatocytes to prevent the nuclear transfer of NF-κB subunits from the cytoplasm [20,21].

The most important target genes of PPAR-α are related to peroxisomal and mitochondrial β-oxidative catabolism of fatty acids, ketogenesis, and nuclear transcription factors linked to inflammation and lipogenesis [22]. PPAR-α activates carnitine palmitoyl-CoA transferase 1α (CPT1A), an importer of fatty acids into mitochondria. It also stimulates more downstream enzymes in the mitochondrial β-oxidation pathway, such as acyl-CoA dehydrogenase medium chain (ACADM) and acyl-CoA dehydrogenase very long chain (ACADVL) [23]. Consequently, PPAR-α fulfills a key role during fasting due to its ability of enhancing cellular energy production through ATP production and ketogenesis [24]. The anti-inflammatory effects of PPAR-α stimulation are attributed to its link with NF-κB and AP-1 [21,25]. Using the carbohydrate-responsive element-binding protein (ChREBP)^−/−^ and PPAR-α knockout mice, it has been shown that PPAR-α cross-talks with ChREBP, a glucose-sensing lipogenic transcription factor, to regulate fibroblast growth factor (FGF)21 expression. The latter is a hepatokine that improves insulin sensitivity and lipid metabolism and controls the preference for sucrose [24,26]. Sterol regulatory element-binding protein (SREBP)1, which is the insulin-sensing variant of ChREBP, is apart from the liver X receptor, which is also regulated by PPAR-α. The chronic activation of PPAR-α in mice results in the upregulation of SREBP1 downstream genes, while this could not be observed in SREBP1^−/−^ mice [23,27]. Consequently, PPAR-α functions as a critical sensor for maintaining cellular energy homeostasis through both catabolic and anabolic pathways.

As shown for PPAR-α, PPAR-δ controls processes related to fatty acid metabolism and inflammation. It is the least well studied PPAR isotype despite of its ubiquitous expression [28]. Using PPAR-δ knockout mice, it could be demonstrated that PPAR-δ exhibits anti-atherogenic properties by reducing very-low density lipoproteins (VLDLs). This is also a consequence from FGF21 signaling [29], which also forms a link to PPAR-α [24,26]. PPAR-δ activation counters angiotensin II-induced adipocyte growth and lipid accumulation. As such, it also reduces the angiotensin II-mediated development of reactive oxygen species, which are implicated in the multiple hit pathogenesis of NASH. Furthermore, the activation of PPAR-δ favors the development of smaller adipocytes, which results in a better adipokine profile. Additionally, the hematopoietic deficiency of PPAR-δ in obese mice attenuates the activation of Kupffer cells that are important for NASH development and stellate cell activation. PPAR-δ also occurs in stellate cells [12,15,26].

PPAR-γ is a critical regulator of adipocyte differentiation and lipogenesis. Chronic stimulation can cause weight gain and obesity, which can eventually lead to related diseases. Hence, PPAR-γ seems to fulfill a developmental function by maximizing energy storage [30]. Paradoxically, PPAR-γ stimulation lessens free fatty acid levels (through adipogenesis), attenuates hepatic glucose production, and increases glucose uptake by the muscles as a result of improved insulin sensitivity [31,32]. Furthermore, PPAR-γ activation reduces inflammation. It attenuates the activation of interferon-γ-stimulated mouse peritoneal macrophages through AP-1, NF-κB, and STAT-mediated mechanisms and blocks tumor necrosis factor-α (TNF-α) production in human monocyte cultures [33,34].

## 3. Dysregulation of PPARs during NASH

The strong correlation between the prevalence of the metabolic syndrome and NAFLD has evoked attention for studying the dysregulation of hepatic PPARs in patients suffering from NASH. Francque and co-workers discovered a correlation between decreased hepatic *PPARA* levels and increased insulin resistance and NASH severity. In addition, a negative correlation with adiponectin levels, an anti-inflammatory adipokine, was measured along with increased visceral obesity. Interestingly, histologic evaluation showed that NASH resolution was associated with an upregulation of *PPARA* together with its target genes, indicating a clear link between PPAR-α function and NASH pathology, thereby opening perspectives for specific PPAR-α-targeted anti-NASH drug development [35]. The expression of the two other PPAR isotypes remained unchanged [35,36]. Nonetheless, a recent study found a correlation between severe, but not moderate, hepatic steatosis and decreased hepatic PPAR-δ mRNA levels. This was accompanied by a decrease in the amount of VLDL receptors in humans. Decreased VLDL receptor levels were as well observed in PPAR-δ knocked-down primary mouse hepatocytes and in the liver of PPAR-δ null mice, confirming the observations in human [29]. Moreover, high-fat diet (HFD)-fed mice show as well decreased hepatic PPAR-δ mRNA levels, invigorating the outcome of the latter study [37].

## 4. PPAR Agonists as Potential Anti-NASH Treatment

### 4.1. PPAR-α Agonists

The interest for the use of PPAR-α agonists (mainly fibrates [38]) in anti-NASH treatment arose already two decades ago, when gemfibrozil was tested in patients suffering from NASH. Gemfibrozil reduced aspartate aminotransferase (AST), alanine aminotransferase (ALT), and gamma-glutamyl transferase (GGT) levels, as well as VLDL triglyceride production. Gemfibrozil also lowered fatty acid mobilization from the adipose tissue and induced lipid clearance in the liver [39]. Fenofibrate treatment in type 2 diabetes patients, suffering from hypertriglyceridemia, decreased serum RANTES, which is also known as the C–C chemokine ligand 5 (CCL5) levels [9], indicating beneficial anti-inflammatory properties related to the NAFLD pathology. Yet, although it improved metabolic syndrome-related parameters, it did not improve liver histology in a 48-week trial with biopsy-proved NAFLD patients. Due to the small trial size of only 16 patients and the lack of a control group [40], further studies should clarify whether fenofibrate might be indicated for anti-NASH therapy. In addition, clofibrate was tested for its possible anti-NASH properties. After one-year treatment, clofibrate did not show any histological improvements in steatosis, inflammation, or fibrosis, nor a reduction in ALT, AST, GGT, bilirubin, or cholesterol, which led to the discontinuation of its evaluation [41]. Pemafibrate, a novel selective PPAR-α agonist, showed to ameliorate liver dysfunction in type 2 diabetes patients, and it was later demonstrated to also improve the NASH pathology in rodents by the stimulation of lipid metabolism and reducing inflammation [42,43].

### 4.2. PPAR-δ Agonists

Activators of PPAR-δ have been mainly investigated for the treatment of dyslipidemia [44,45,46,47]. One selective PPAR-δ agonist, named seladelpar, was recently suspended from a phase II trial due to unexpected histological findings. Nevertheless, seladelpar improved the serum lipid profile in dyslipidemic patients and also reduced liver enzyme levels [44,45]. In a foz/foz mouse model, seladelpar also reversed the hepatic storage of lipotoxic lipids and improved insulin sensitivity and serum lipid profile, indicating beneficial properties for treating NASH [48].

### 4.3. PPAR-γ Agonists

A multitude of studies has evaluated the use of insulin-sensing agents for the treatment of NASH [49,50]. Thiazolidinediones, which are PPAR-γ agonists, are such a class of insulin sensing drugs [30,51]. The potential anti-NASH efficacy of pioglitazone (and vitamin E) has been evaluated in the so-called PIVENS (Pioglitazone, Vitamin E, or Placebo for Nonalcoholic Steatohepatitis) trial. Here, it was found that pioglitazone and vitamin E reduce hepatic steatosis and lobular inflammation, but not concomitant fibrosis (Table 1) [52].

Vitamin E alone met the primary outcome, which was a histologic improvement of NASH based on a scoring system [52,54]. Nevertheless, in a later trial, Cusi and co-workers observed the resolution of NASH in 51% of the studied subjects suffering from prediabetes and type 2 diabetes with NASH upon long-term pioglitazone treatment [55]. Pioglitazone also improved liver injury and fibrosis in non-diabetic NASH patients [56]. As a result, the American Association for the Study of Liver Diseases (AASLD) and the European Association for the Study of the Liver (EASL) now recommend the use of pioglitazone and vitamin E for the treatment of biopsy-proven NASH. Albeit, it should be noted that the use of pioglitazone in subjects that do not suffer from type 2 diabetes is off-label and that thiazolidinediones typically cause weight gain [57,58,59]. Furthermore, pioglitazone was shown to cause a 63% increased risk for developing bladder cancer in a population-based cohort study. This was not the case for rosiglitazone, another thiazolidinedione that is/was also administered as an insulin sensitizer to type 2 diabetes patients [60]. Unfortunately, in a two-year follow-up study, rosiglitazone did only show anti-steatogenic effects in the first year of treatment, nor additional anti-NASH effects upon longer administration [61,62]. Yet, long-term rosiglitazone treatment has been associated with an increased risk of myocardial infarction and heart failure, although without influencing cardiovascular mortality [63]. Therefore, rosiglitazone has been withdrawn from the market in Europe, and its use is highly restricted in the US [64]. Lobeglitazone, one of the latest developed PPAR-γ agonists, has also been evaluated in type 2 diabetes patients suffering from NAFLD (Table 1). Twenty-four-week treatment improved glucose homeostasis, lipid profile, and hepatic steatosis, with a moderate weight gain in comparison to pioglitazone. Decisive anti-NASH efficacy of lobeglitazone should be assessed in further randomized controlled trials using liver histology as a primary endpoint [51,65,66].

### 4.4. PPAR-α/δ Agonists

Elafibranor is currently under clinical phase III evaluation and one of the most promising anti-NASH drugs. It is a first-in-class dual PPAR-α/δ agonist that has shown to resolve NASH after a 52-week treatment by means of reduced liver enzymes, steatosis, and markers of systemic inflammation. Furthermore, a reduction in fibrosis could be observed as well compared to the non-responders. It does not cause weight gain [67,68].

### 4.5. PPAR-α/γ Agonists

Given the overlapping and distinct metabolic effects of the thiazolidinediones and fibrates, drugs targeting both receptors were thought to be ideal for treating diseases related to the metabolic syndrome [18,69]. Only one dual PPAR-α/γ agonist has been (experimentally) tested for the treatment of NASH. Saroglitazar, which is already authorized in India for the treatment of diabetic dyslipidemia [70], has predominant PPAR-α activity but also agonizes PPAR-γ. This compound induced a reduction of NASH characteristics in both in vitro (palmitate-exposed HepG2 and HepG2-LX2 co-cultures) and in vivo (choline-deficient HFD-fed mice) models, where it seemed to be more effective than pioglitazone and fenofibrate [71]. Saroglitazar is currently being evaluated in a clinical phase II study (Table 1), but no clinical results have been divulgated. Several (older) PPAR-α/γ agonists exist, but much less is known about their potential anti-NASH efficacies. A major issue of this drug class, despite improving glucose and lipid metabolism, is the development of multiple adverse effects, such as edema, cardiovascular events, weight gain, renal dysfunction, and abnormal liver enzyme tests. Their further development has consequently been discontinued [72,73].

### 4.6. PPAR-pan Agonists

Lanifibranor, one of the most recently developed potential anti-NASH drugs, is the first PPAR-pan agonist, targeting all three PPAR isotypes. Lanifibranor was found to reduce liver steatosis, inflammation, and hepatocyte ballooning in different mouse models of NASH (methionine- and choline-deficient (MCD) diet-fed and foz/foz mice). An attenuated fibrotic response was also observed in the MCD diet-fed mouse model and CCl_4_-induced fibrosis in mice. In addition, it showed the inhibition of fibrotic genes in HFD-fed mice and inhibited the activation and proliferation of primary human hepatic stellate cells [74].

## 5. Strategies for Improving PPAR-Targeted Anti-NASH Drug Testing and Therapy

Based on the lack of an approved anti-NASH therapy and the current models that are used, it can be argued that (i) current disease models fail in representing the heterogeneity of the disease, and as such only illuminate a small facet of the NAFLD pathology, (ii) targeting nuclear receptors with broad and diffuse working mechanisms might not be sufficient for treating NASH, and (iii) personalized targeting of the underlying comorbidities might be the key adjuvant therapy in a heterogeneous population of NASH patients.

### PPAR-Targeted Preclinical Drug Testing

From the abovementioned studies, it is clear that murine models can significantly contribute to the assessment of potential anti-NASH characteristics of novel drugs targeting different PPAR isotypes. Nevertheless, these agents could be tested in a more efficient way when interspecies differences regarding (i) the PPAR biology, (ii) responses to PPAR agonism, and (iii) specific polymorphisms related to NASH pathology could be taken out of the equation.

**Figure 2:**
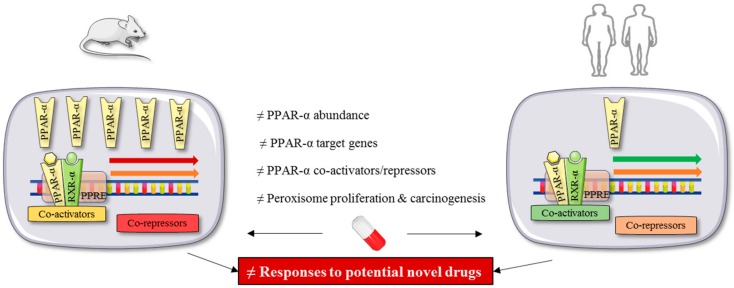
Discrepancies in PPAR biology between rodents and humans may result in different outcomes during drug testing. Hepatic PPAR-α is higher expressed in rodents than in humans. It also has different target genes and coactivators/repressors. Consequently, drugs that target PPAR-α can show different efficacies in rodent- and human-based studies.

It is estimated that primary rodent hepatocytes express 10 to 20 times higher levels of PPAR-α than observed in human cells [75]. Furthermore, rodent and human PPAR-α show differences in responsiveness toward PPAR agonists [76]. For example, the exposure of HepG2 and CV-1 cells transfected with the human or rat PPAR-α to PPAR-α agonists (clofibrate, ETYA, and WY-14) show differential levels of PPAR-α activation [77]. On top, endogenous PPAR-α ligands differentially activate the mouse and human PPAR-α in transfected COS-7 cells. This might, apart from the higher basal PPAR-α abundance in mouse liver, explain why rodents develop liver cancer upon the administration of fibrates, which does not occur in humans [76,78]. Such differences are of major importance, since the PPAR isotypes need to be agonized in a specific manner and not only in the most potent way possible. This seems rather impossible to evaluate when only rodent models are being used (Figure 2).

This issue was addressed by Yang and co-workers, who generated PPAR-α humanized transgenic mice by using a P1 phage artificial chromosome (PAC) genomic clone bred onto a PPAR-α-null mouse background. When fenofibrate was administered to these transgenic mice, they did not show hepatomegaly and hepatocyte proliferation compared to wild-type mice. In addition, the miRNA MIRLET7C, which is involved in cell growth and regulation of the proto-oncogene MYC, was differentially regulated between the transgenic and wild-type mice, indicating a divergent regulation of the rodent and human PPAR-α. However, the authors pointed to the fact that these transgenic mice still show peroxisome proliferation upon fenofibrate administration. Yet, they did not take into account the possible regulatory differences of coactivators/repressors of PPAR-α, rendering unique responses to (partial) PPAR agonists [17,79]. This concern was later addressed by Tateno and co-workers, who created a mouse model, possessing a humanized liver containing more than 70% human hepatocytes and expressing more than 82% of the human genes. It could be shown that fenofibrate did not induce peroxisome proliferation in humanized livers compared to wild-type mice. It was assumed that human coactivators/repressors regulate the action of PPAR-α in a humanized way [80,81]. Later, it was shown that PPAR-α activation in human hepatocytes occurred in a more moderate way than in the residual mouse hepatocytes, confirming the abovementioned hypotheses [82].

Apart from these sophisticated refinements in animal experimentation and successful application, more practical tools exist to test the efficacy of new compounds with potential anti-NASH efficacy. Rogue et al. tested a battery of PPAR agonists (troglitazone, rosiglitazone, muraglitazar, tesaglitazar, fenofibrate, and bezafibrate) in oleic acid-overloaded HepaRG cells. Only troglitazone did not reduce the oleic acid-induced increased lipid load, demonstrating the potential of this model for the preclinical assessment of new anti-NAFLD compounds [83]. Yet, although harboring great applicability potential, none of the successfully tested drugs in the in vitro model showed convincing results during clinical studies [40,61]. Here, one should keep in mind that the complexity and inflammatory aspects of the NASH pathology are absent in the above-mentioned in vitro system. Feaver and co-workers constructed a human co-culture model containing primary human hepatocytes, stellate cells, and macrophages in a lipotoxic medium consisting of elevated insulin, glucose, and fatty acid levels [84]. Elafibranor lowered the lipid load in this system and attenuated the inflammatory response by downregulating interleukin (IL)-6 and CCL2 secretion [85], which correlates with clinical observations [67]. Our group could recently make similar observations using a human-derived adult stem cell-based model of NASH induced by fatty acids, insulin, glucose, and inflammatory cytokines. In this in vitro model, elafibranor potently reduced the increased lipid load and attenuated the inflammatory response [86].

**Figure 3:**
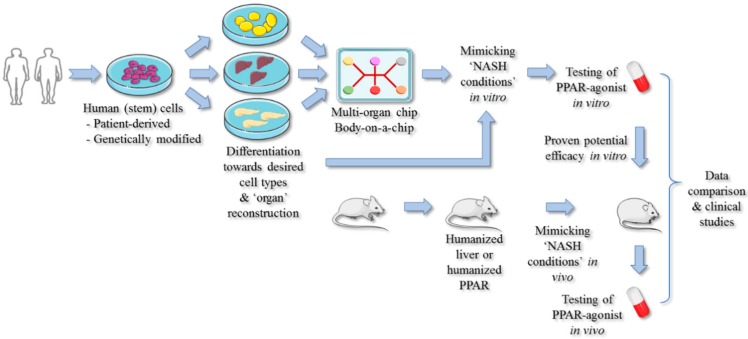
The implementation of human-based systems may result in reliable PPAR-targeted drug testing. To avoid inter-species-related discrepancies, human (stem) cells can be used to evaluate PPAR agonists and assess their potential anti-NASH efficacies. Multiple cell types can be co-cultured and/or interconnected to better represent whole-body metabolism. In addition, humanized mice can be employed to further evaluate the anti-NASH properties of novel compounds.

The lack of approved (PPAR-targeted) therapies against NASH is at least partly ascribed to the lack of validated in vitro and in vivo models. Major issues are important interspecies differences and a relatively high cost [87]. Furthermore, given the multi-regulatory functions and broad tissue distribution of PPARs, it is clear that studies using non-human-based species should be interpreted with caution. Mice hosting humanized hepatic PPARs-α [79] or liver [81] address this issue only partly, since NAFLD is a multi-organ disease. In addition, PPAR(-α) target genes may be different between humans and rodents [23]. Therefore, we believe that human-based in vitro models could greatly contribute to select compounds with suitable PPAR isotype activity as well in simple [86,88] as in more complex co-cultures [84,85]. These models could ultimately be fine-tuned to specific ‘NASH subtypes’, mimicking the heterogeneity within the population of the molecular driving mechanisms of the disease and being suitable to evaluate different (combination) therapies. The lack of multiple cell types and/or organs for testing novel drug candidates as PPAR agonists is a drawback of most in vitro models. Yet, the advancements made to the development of ‘human-on-a-chip’ models seem promising [89]. Although still much has to be realized [90], the interconnection of in vitro surrogate organs such as liver, pancreas, and adipose tissue, merged with data of humanized animal models, could enable more reliable PPAR-targeted anti-NASH drug testing and thus improve clinical outcomes. Furthermore, by implementing in these chips patient-derived (induced-pluripotent) stem cells carrying high-risk genetic backgrounds for developing NASH, the assessment of personalized therapies might ever become a reality (Figure 3) [91].

## 6. Targeted PPAR Agonism as NASH-Specific Therapy

Only a minority of steatosis subjects progresses toward NASH, indicating that environmental, hereditary, or other secondary causes (e.g., certain infections and extrahepatic conditions) lay at the basis of NAFLD progression [92]. This has also been outlined in the ‘multiple hit hypothesis’ for the pathogenesis of NASH [93]. In addition, the moderate success rates that have been obtained so far during clinical trials raise the question whether the current strategies for anti-NASH therapy are sufficiently addressing the complexity and the diverse pathogeneses of NASH [10]. It seems reasonable to conclude that PPAR-targeted anti-NASH therapy should preferably also consider the specific underlying etiologies and causative factors of the disease.

### 6.1. Diet-Induced NASH

Nutritional habits are often uniformly classified as a major cause of the metabolic syndrome and obesity. Nevertheless, different carbohydrates and lipid species can govern and drive divergent metabolic processes [94,95]. However, this is often neglected in the development of novel drugs against diseases with heterogeneous pathogeneses.

A high-carbohydrate diet in combination with low-energy expenditure induces de novo lipogenesis that results in the generation of fatty acids and a variety of lipid species. Glucose is generally regarded as the main sugar that drives de novo lipogenesis. Yet, fructose is also widely present in many drinks and foods and can induce as well de novo lipogenesis and fat accumulation [96]. Fructose may activate PPAR-γ coactivator-1β, which is a coactivator of SREBP-1c, triggering the lipogenic cascade [97]. It has also been reported in rat studies that fructose inhibits the function of PPAR-α, resulting in decreased β-oxidation and increased NF-κB activity [98,99]. Fructose may activate an inflammatory response due to its specific metabolism that only slightly differs from glucose catabolism. Glucose is first metabolized by glucokinase/hexokinase, while fructose is metabolized by fructokinase C. The latter enzyme phosphorylates fructose, followed by the formation of glyceraldehyde and dihydroxyacetone phosphate by the action of aldolase B. The persistent phosphorylation of fructose requires ATP and phosphate, resulting in the deposition of uric acid, which acts as a danger-associated molecular pattern (DAMP) [95]. DAMPs on their turn activate inflammasomes to produce IL-1β, which triggers an inflammatory response and thereby lay at the basis of NASH [100]. PPAR agonism for fructose-driven NASH might prove effective because of the specific action of fructose on PPAR(-α) activity. Nonetheless, it is unlikely that PPAR-α agonism can also capture the unique metabolism of fructose that results in the formation of uric acid that in turn drives the inflammatory reaction. The deposition of uric acid might remain present, resulting in a lean but inflamed liver.

Moreover, serum uric acid levels in NAFLD patients have been proposed as potential predictors of liver damage severity [101]. It has been shown in vitro, using HepG2 cells, that fructose induces lipid accumulation and uric acid formation. This is prevented by allopurinol [102], which is a drug that is indicated for the treatment of gout. In this view, PPAR-(γ) agonists with uricosuric properties could be eminent anti-NASH drugs. For example, arhalofenate, a selective partial PPAR-γ modulator, has been developed as a lipid-lowering drug, yet has also been found to block the reabsorption of uric acid through the inhibition of URAT1 in the proximal tubules of the kidney, thereby lowering serum uric acid levels [103,104]. African Americans show the lowest NAFLD prevalence among different ethnicities and exhibit fructose malabsorption, possibly rendering hepatoprotective effects [105,106].

### 6.2. Obesity and Type 2 Diabetes-Induced NASH

Obesity increases the risk for developing NAFLD and metabolic syndrome-related complications, in which the waist circumference is a key determinant [106]. In obesity, white adipose tissue produces a plethora of inflammatory mediators [107], leading to low-grade chronic inflammation. This effect is mediated by adipose tissue macrophages secreting pro-inflammatory mediators (TNF-α, IL-6), and detrimentally affecting insulin signaling in adipose tissue and liver [108]. TNF-α, but also lipotoxic lipid species such as free fatty acids, provoke the phosphorylation of serine residues of the insulin receptor substrate 1, which prevents signal transmission from the insulin receptor [107,109]. As such, inflammation in obesity-driven NAFLD is mainly derived from adipose tissue, instead of the liver. Impressively, the bariatric surgery of morbidly obese NASH patients resolved NASH in up to 85% of the patients [5], and it has been further observed that TNF-α levels decline with weight loss [110,111]. This points to the fact that adipose tissue is a major driver of the disease.

PPAR-γ agonists can reduce obesity-induced inflammation and improve insulin signaling [21]. Yet, the use of (full) PPAR-γ-agonists is limited because of weight gain [30]. Although partial PPAR-γ agonism could be efficient for obesity-induced NASH, it is clear that weight loss through lifestyle modification, bariatric surgery, or pharmacological therapy is of major importance in this subgroup of NASH patients [4,5]. It efficiently may reduce the source of inflammation and (whole-body) insulin resistance.

### 6.3. Lean NASH

Ethnicity is another risk factor for NAFLD. Several studies reported that a single nucleotide polymorphism (SNP) in PNPLA3 (rs738409) confers a higher susceptibly for developing NASH [112,113,114]. As this genetic trait is most common in Hispanics, they carry the highest risk. On the contrary, African Americans are the least susceptible for developing NAFLD [107,112].

PNPLA3 or adiponutrin is an adipose triglyceride lipase in which the rs738409 variant structurally prevents substrate binding [115]. The PNPLA3 rs738409 variant does not modify the disease through changes in insulin sensitivity, metabolic syndrome, body mass index, or dyslipidemia [116,117,118]. This subtype of NASH patients would theoretically not benefit from insulin-sensing PPAR agonists, yet they might do from PPAR agonists with strong β-oxidative capacity. On the contrary, the loss-of-function SNP Gly972Arg in the insulin receptor substrate-1 diminishes insulin signaling and increases the susceptibility for NAFLD progression [119]. Therefore, insulin sensitizing agents could be indicated as anti-NASH therapy when carrying this specific SNP [120]. However, lean NAFLD is not only caused by genetic alterations. Up to 5% of the Western population is ‘metabolically obese’, in which insulin resistance, hypertriglyceridemia, and hyperuricemia occurs [121]. In contrast to some (e.g., PNPLA3 rs738409) genetically-driven lean NASH patients, ‘metabolically obese’ NASH patients might favor from insulin-sensing PPAR(-(α)/γ) agonists. Indeed, 80% of the non-obese type 2 diabetes patients exhibit liver steatosis [122]. Other causes of lean NASH, such as HIV treatment, endocrine disorders (e.g., polycystic ovarian syndrome, hypothyroidism), total parenteral nutrition, jejunoileal bypass, or the use of NASH-inducing drugs (e.g., valproic acid, amiodarone, and methotrexate) [106,123,124], all ask for the individual identification and evaluation of the specific NASH pathogenesis and potential subsequent therapy.

### 6.4. Microbiome-Induced NASH

The microbiome is a highly dynamic population of microbiota subject to nutrition, environment, and immunity [125]. Alterations in composition in this often-called ‘forgotten organ’ can lead to dysbiosis, which may result in a plethora of diseases [126,127]. NAFLD has been associated with an increased gut permeability, small intestinal bacterial overgrowth [128], and endotoxemia (in which lipopolysaccharide (LPS) plays a prominent role) [129,130]. LPS is a component of Gram-negative bacteria, which are elevated in the gut of NASH patients [131]. LPS affects insulin signaling through the Toll-like receptor 4–monocyte differentiation antigen CD14 system, leading to insulin resistance [132]. It also holds the capacity to trigger inflammasome activation, leading to sustained hepatic inflammation [133]. Much more complex alterations in the gut microbiome of NASH patients have been reported [125,134], but it is clear that PPAR-targeted therapy might not be effective in the long-term for these patients, since the real cause of the disease is not targeted. Multiple trials are running to specifically target and restore the gut microbiome (e.g., using probiotics) in NASH patients, in which already satisfactory results have been obtained [135].

## 7. Outlook and Conclusions

A multitude of agents targeting one, two, or all three PPAR isotypes has been evaluated and is under evaluation for anti-NASH treatment. Although theoretically promising, none of the on-the-market anti-hyperglycemic and anti-hyperlipidemic drugs have so far proven to adequately tackle NASH. Hitherto, elafibranor, currently being evaluated in a clinical phase III trial, seems to pose the best outbalanced activity on the PPAR-α and -δ isotypes to treat NASH [67]. Nonetheless, two other PPAR agonists, lanifibranor [74] and saroglitazar [71], respectively a PPAR-pan and PPAR-α/γ agonist, are both being tested in clinical phase II trials and seem promising as well.

**Figure 4:**
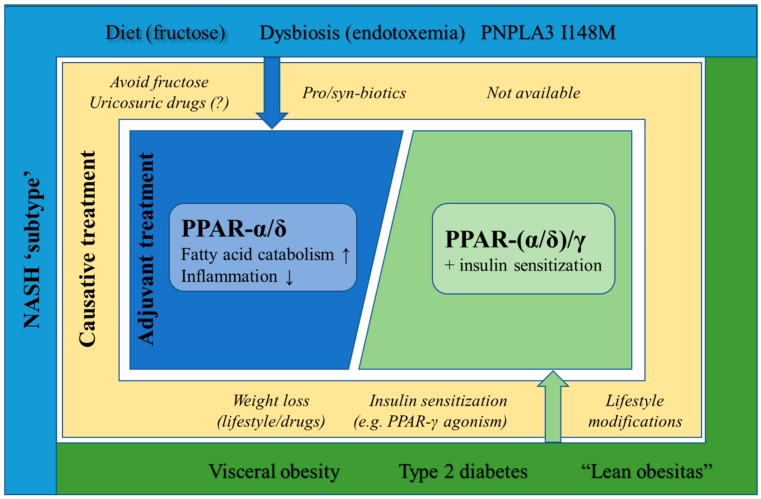
Distinct PPAR-targeted anti-NASH therapies could be applied to different NASH etiologies. PPAR agonists are able to attenuate different features of NASH. Therefore, different PPAR-targeted therapies could be administered to NASH patients following their specific etiologies.

As the origin of NASH is not as clear-cut as previously thought [136,137], and NASH patient trials are generally obtaining unsatisfactory low success rates [10], the rationale behind the development of novel anti-NASH drug candidates and/or combination therapies could perhaps benefit from a subdivision into ‘NASH types’, paralleling the ‘multiple hit hypothesis’ for the pathogenesis of NASH [93] (Figure 4).

The PPAR agonists under development all target consequences of pre-existing, underlying morbidities, rendering them the potential to rapidly intervene in case of liver steatosis and inflammation. Yet, apart from the often insulin-sensing effects of these compounds, no other disease drivers and/or modifiers are targeted, leaving the cause behind NAFLD progression untouched. Therefore, it can be questioned whether subdividing NASH and diagnosing into subtypes could not increase the efficiency to target the causes that lay at the basis of the disease state and improve clinical outcomes. Furthermore, it is plausible that combination therapies, targeting different aspects of the disease (e.g., probiotics together with a PPAR agonist), are necessary to both target the initial disease driver and relieve the existing hepatic steatosis and inflammation. Indeed, combination therapies are already being explored to also target uncovered disturbed pathways. This has been outlined by the Food and Drug Administration as well [138]. Although elafibranor seems to be one of the most promising compounds for anti-NASH treatment, a clinical program to evaluate combination therapies with a glucagon-like peptide-1 receptor agonist and a sodium-glucose transporter-2 inhibitor has been recently announced [139]. Nevertheless, the transition of benign hepatic steatosis to NASH could be delayed or prevented by PPAR-(α/δ)-agonists. By lowering the hepatic lipid load at the early steatosis stage, the liver might regain physiological functions before adaptive mechanisms are exhausted.

These considerations in anti-NASH therapy could be translated into the early drug developmental stages, in which human-based approaches can provide information with respect to potential efficacy. The main reason behind the use of human-based models in PPAR-targeted anti-NASH drug development is connected with the important inter-species differences in the biology of PPARs [86,140]. Yet, mice carrying humanized (hepatic) PPARs could be key for the evaluation of multi-organ targeted anti-NASH therapies [80,81]. In the future, human-based body-on-a chip methodologies might be considered as suitable human-relevant test models [86,89,91].

## Figures and Tables

**Table 1:** Evaluation of peroxisome proliferator-activated receptors (PPAR) agonists for anti-non-alcoholic fatty liver disease (NAFLD) treatment in clinical trials. ALT: alanine aminotransferase, NASH: non-alcoholic steatohepatitis.

Agent	PPAR Isotype	Primary Outcome	Duration	Status	Trial Number/Reference
Phase 4					
Lobeglitazone	α/γ	Changes from baseline in controlled attenuation parameters	24 weeks	Completed (no results posted)	NCT02285205 [51]
Rosiglitazone Alpha-lipoic acid Rosiglitazone + alpha-lipoic acid	γ	Improvement in NASH histological scoring system	24 weeks	Terminated (because of withdrawal of Avandia sale due to its risks outweigh its benefits)	NCT01406704
Phase 3					
Elafibranor	α/δ	NASH resolution without worsening of fibrosis	72 weeks 4 years	Recruiting	NCT02704403
Pioglitazone Vitamin E	γ	Change in standardized scoring of liver biopsies	96 weeks	Completed (has results)	NCT00063622 [52]
Phase 2					
Lanifibranor	α/δ/γ	Decrease of at least two points in SAF (Steatosis, Activity, and Fibrosis) activity score combining hepatocellular inflammation and ballooning without the worsening of fibrosis	24 weeks	Active	NCT03008070
Lifestyle-intervention Pioglitazone Berberine	γ	Improved metabolic parameters (glucose, lipid, liver enzymes, etc.,).	16 weeks	Completed (results submitted)	NCT00633282 [53]
Pioglitazone	γ	Reduction in the NASH activity index by three points or more with improvements of at least one point each in steatosis, parenchymal inflammation, and hepatocellular injury	48 weeks	Completed (has results)	NCT00062764
Pioglitazone	γ	Improvement in hepatic histology as determined by the reduction of at least three points in the NASH activity score	48 weeks	Completed (no results posted)	NCT00013598
Saroglitazar	α/γ	Percentage change from baseline in serum ALT levels	16 weeks	Active	NCT03061721
Saroglitazar magnesium	α/γ	Safety measured by adverse events, vital signs, physical exams, body weight, electrocardiograms, and lab results	24 weeks	Recruiting	NCT03639623
Seladelpar	δ	Evaluation of the hepatic fat fraction, safety, and tolerability in NASH patients	12 weeks	Suspended (unexpected histological findings)	NCT03551522
